# Spherical coral-like synovial chondroma within a popliteal cyst: a case report

**DOI:** 10.3389/fmed.2025.1670296

**Published:** 2025-10-17

**Authors:** Bingyan Mao, Qi Peng, Jicai Li, Zuoquan Qin, Shenke Xie

**Affiliations:** The People′s Hospital of Shimen County, Changde, China

**Keywords:** synovial chondromatosis, synovial chondroma, spherical coral morphology, popliteal cyst, Baker’s cyst, case report, knee joint

## Abstract

Popliteal cysts are common lesions, primarily characterized by soft tissue swelling, which may occasionally present with calcifications or small osteochondromas, and can rarely lead to restricted movement. We report a case involving a spherical coral-like synovial chondroma located within a popliteal cyst, which caused significant pain and limited the flexion and extension of the knee joint. Notably, the appearance of the synovial chondroma was strikingly similar to that of spherical coral, and its distinctive imaging features are previously unreported in the literature. Following the excision of both the popliteal cyst and the intra-cystic spherical coral-like synovial chondroma, the patient’s range of motion in the knee joint returned to normal. The unusual morphology may arise from constrained growth within the cyst under pressure during joint movement, potentially influenced by the hyperuricemic microenvironment. Clinicians should consider synovial chondromatosis in the differential diagnosis of complex or calcified popliteal cysts.

## Introduction

Popliteal cysts, or Baker’s cysts, represent common benign fluid collections within the popliteal fossa, frequently associated with underlying knee joint pathology such as osteoarthritis, meniscal tears, or chronic synovitis (1–3). These cysts typically arise from the gastrocnemius-semimembranosus bursa, which often communicates with the knee joint. While most cysts are straightforward to diagnose, the presence of calcifications or ossifications within them complicates the differential diagnosis, potentially mimicking calcified soft tissue tumors (e.g., liposarcoma, synovial sarcoma) or vascular anomalies (e.g., aneurysm, AVM) (1, 2). Treatment ranges from conservative management to surgical excision.

Synovial chondromatosis (SC) is an uncommon benign proliferative disorder of the synovium, characterized by the formation of cartilaginous nodules that may detach as loose bodies. It predominantly affects the knee joint in individuals over 40 years, with a male predilection (4, 5). Etiology remains unclear, but trauma, chronic irritation, and degenerative joint disease are implicated factors (6–8). The occurrence of SC specifically within a popliteal cyst is exceedingly rare. These studies did not describe the appearance of synovial chondroma in popliteal cysts. However, the images in the articles depicted irregular, calcified free bodies (9–11). Notably, a synovial chondroma exhibiting a distinct spherical coral-like morphology has not been previously reported in the literature. We present such a case, detailing its clinical presentation, unique imaging features, pathological characteristics, surgical management, and outcome, while discussing potential pathogenic mechanisms, including the possible role of the patient’s hyperuricemia.

## Case report

### Patient information and history

The patient, a 66-year-old female, was admitted to Shimen County People’s Hospital (Changde, China) on January 27, 2025, for further examination and treatment due to left knee joint pain persisting for over 10 years, along with the recent discovery of a left popliteal fossa mass that has been accompanied by aggravated pain and limited mobility for the past half month. The patient provided consent the publication of this study and the study received approval from the Ethics Committee of Shimen County People’s Hospital.

### Clinical findings

Physical examination revealed slight swelling in the left popliteal fossa, with no redness or swelling of the surface skin, and the local skin temperature was normal. A 6 × 4 cm mass was palpable, exhibiting a firm texture with clear boundaries, no obvious adhesion, local tenderness, and no significant mobility. There was no radiating pain in the left calf, and no pulsation or vascular murmur was detected upon auscultation. The range of motion for flexion and extension was 0-10-105°. The patellar ballotment test was positive, patellar mobility was good, and the patellar grind test, hyperextension test, and hyperflexion test were all positive as well. The McMurray test was positive, while the lateral stress test and anterior and posterior drawer tests were negative, and the Lachman test was positive, with the rotational test negative. Pain worsened during left knee flexion, and there was no lymphadenopathy in the left popliteal fossa or inguinal region.

### Diagnostic assessment

Blood supply and sensation in the left lower limb were normal. Laboratory tests indicated elevated serum creatinine 94 μmol/L (reference range 41–81 μmol/L), uric acid 548 μmol/L (reference range 155–357 μmol/L), and blood glucose 10.4 mmol/L (reference range 3.9–6.1 mmol/L). Complete blood count, blood alkaline phosphatase, rheumatoid factor, and high-sensitivity C-reactive protein (hs-CRP) were all within normal ranges.

**Figure 1:**
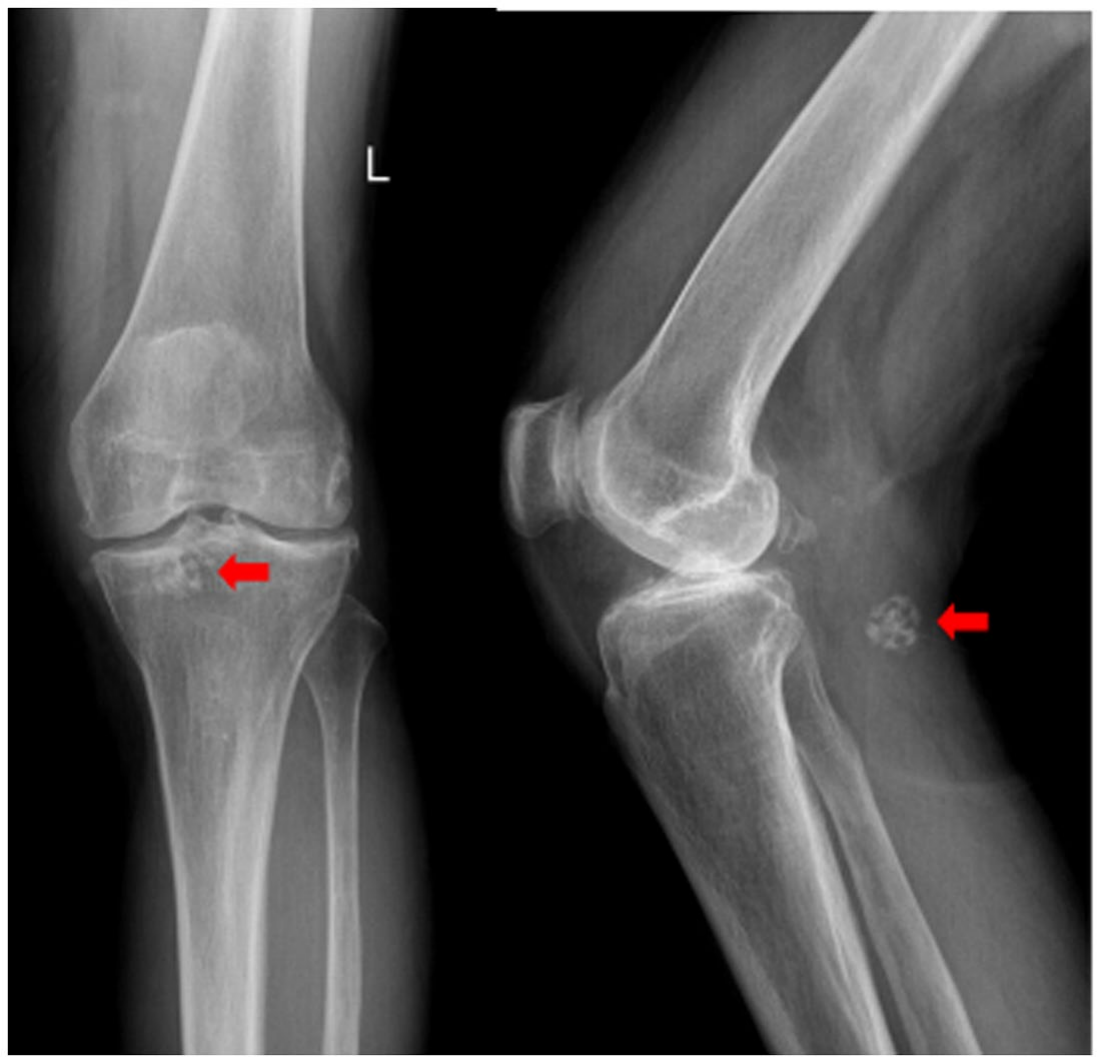
Anteroposterior and lateral radiographs of the left knee. Degenerative changes are evident. A spherical coral-like calcified nodule is indicated by the red arrow.

The imaging examination results revealed that the X-ray images demonstrated the presence of osteophyte shadows at the margins of the bones constituting the left knee joint, with osteophytes having formed in certain areas. Additionally, the tibial intercondylar eminence exhibited a pointed morphology, the joint space was significantly narrowed, and the subchondral bone displayed signs of sclerosis. In the popliteal region of the left knee, a spherical coral-like calcified nodular shadow approximately 16 mm in diameter was observed (Figure 1).

**Figure 2:**
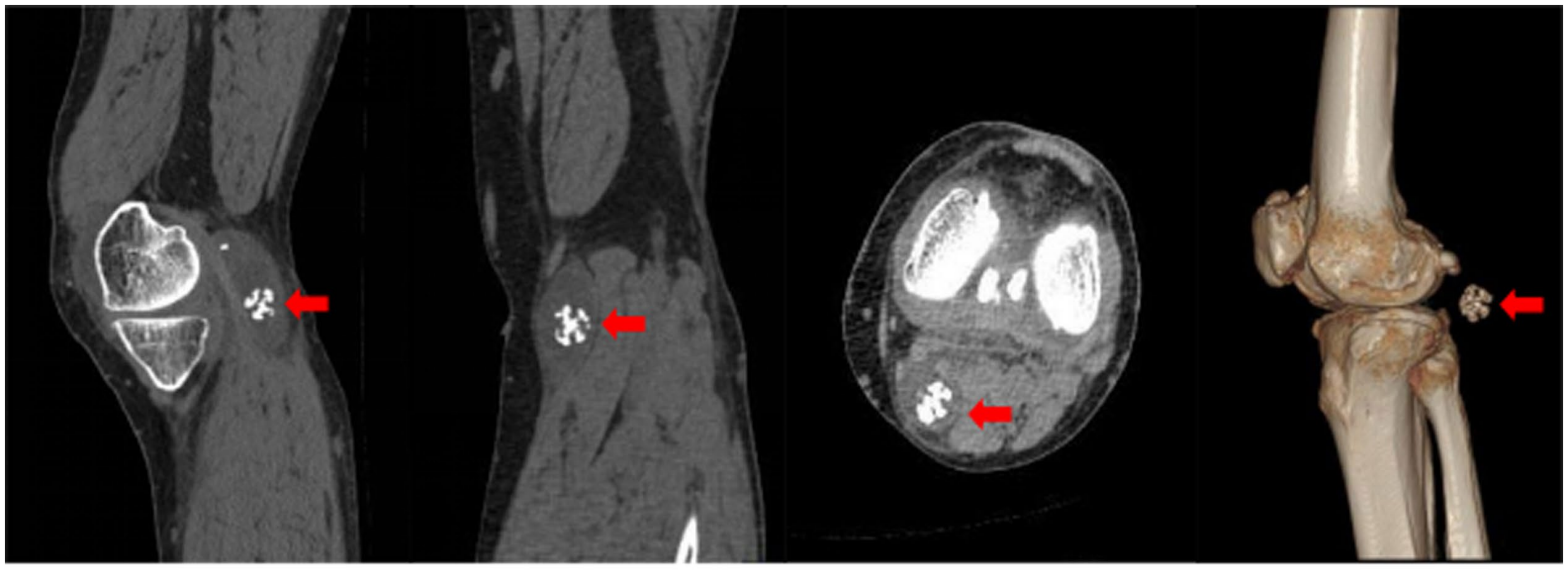
Axial CT image of the knee. The red arrow highlights the spherical coral-like synovial chondroma within the hypodense popliteal cyst.

CT scans revealed that the intercondylar eminences of both tibias and the upper margins of the patellas appeared sharpened. Osteophytes were observed at the lower ends of the femurs and the upper ends of the tibias, accompanied by significant narrowing of the joint spaces in both knees. Effusions were noted in the suprapatellar bursae of both knees, with a more pronounced presence in the left knee. An oval-shaped hypodense cystic lesion measuring approximately 60 mm × 25 mm was identified in the popliteal fossa of the left knee, containing multiple nodular masses; the largest of these had a diameter of about 20 mm and resembled a spherical coral (Figure 2).

**Figure 3:**
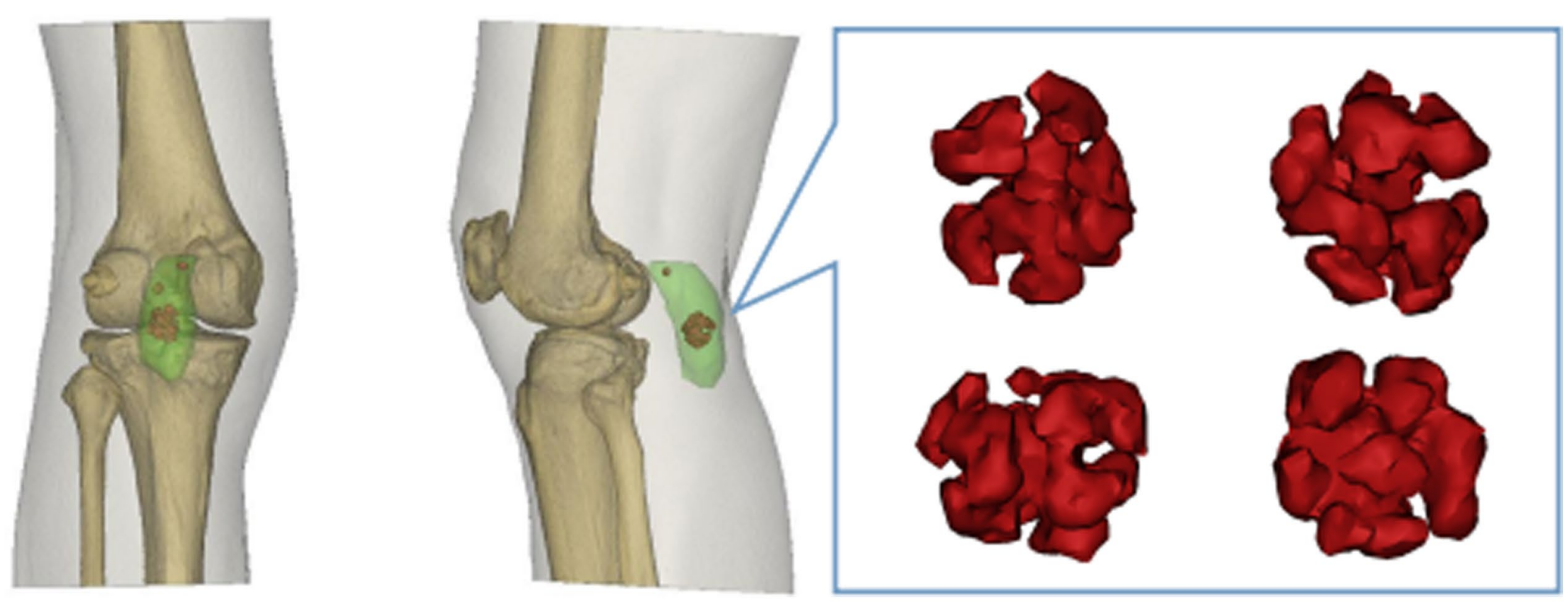
Three-dimensional CT reconstruction of the knee. Light green rendering represents the popliteal cyst. Red rendering depicts the intracystic synovial chondroma exhibiting its spherical coral-like structure.

Furthermore, the CT slice DICOM data were imported into Mimics 21.0 software for 3D reconstruction, which revealed four nodular hyperdense masses of varying sizes within the hypodense cystic lesion in the popliteal fossa, with the largest mass also resembling a spherical coral (Figure 3).

**Figure 4:**
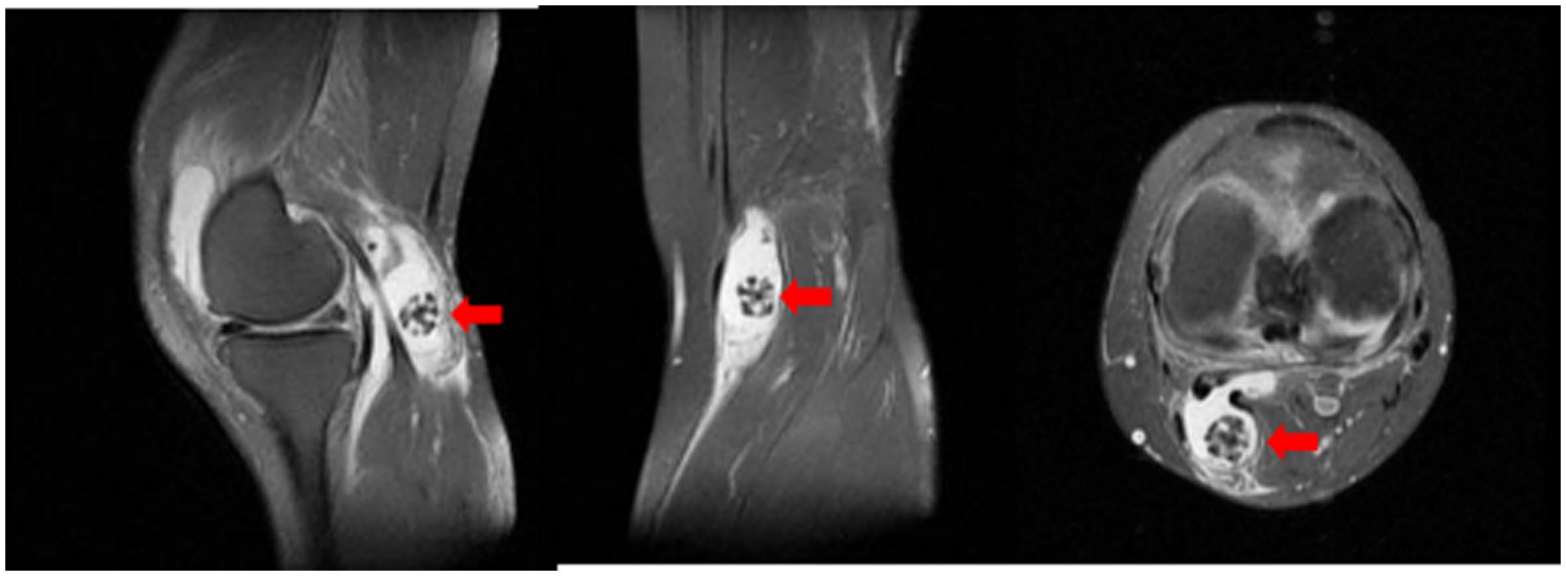
Sagittal T2-weighted MRI of the knee. The red arrow points to the spherical coral-like synovial chondroma within the popliteal cyst (hyperintense fluid signal). Associated joint effusion and degenerative changes are visible.

MRI examination revealed the presence of a synovial cyst in the popliteal fossa, accompanied by joint effusion and an intracystic mass exhibiting a spherical coral-like morphology. The synovial cyst was found to communicate with the joint cavity, and significant fluid accumulation was noted in the suprapatellar bursa. Additionally, tears were identified in the anterior and posterior horns of both the medial and lateral menisci. Subchondral bone marrow edema and cystic changes were observed beneath the articular surfaces of the distal femur and the posterior margin of the patella, with damage also noted in the articular cartilage surfaces (Figure 4).

### Therapeutic intervention

#### Surgical treatment

**Figure 5:**
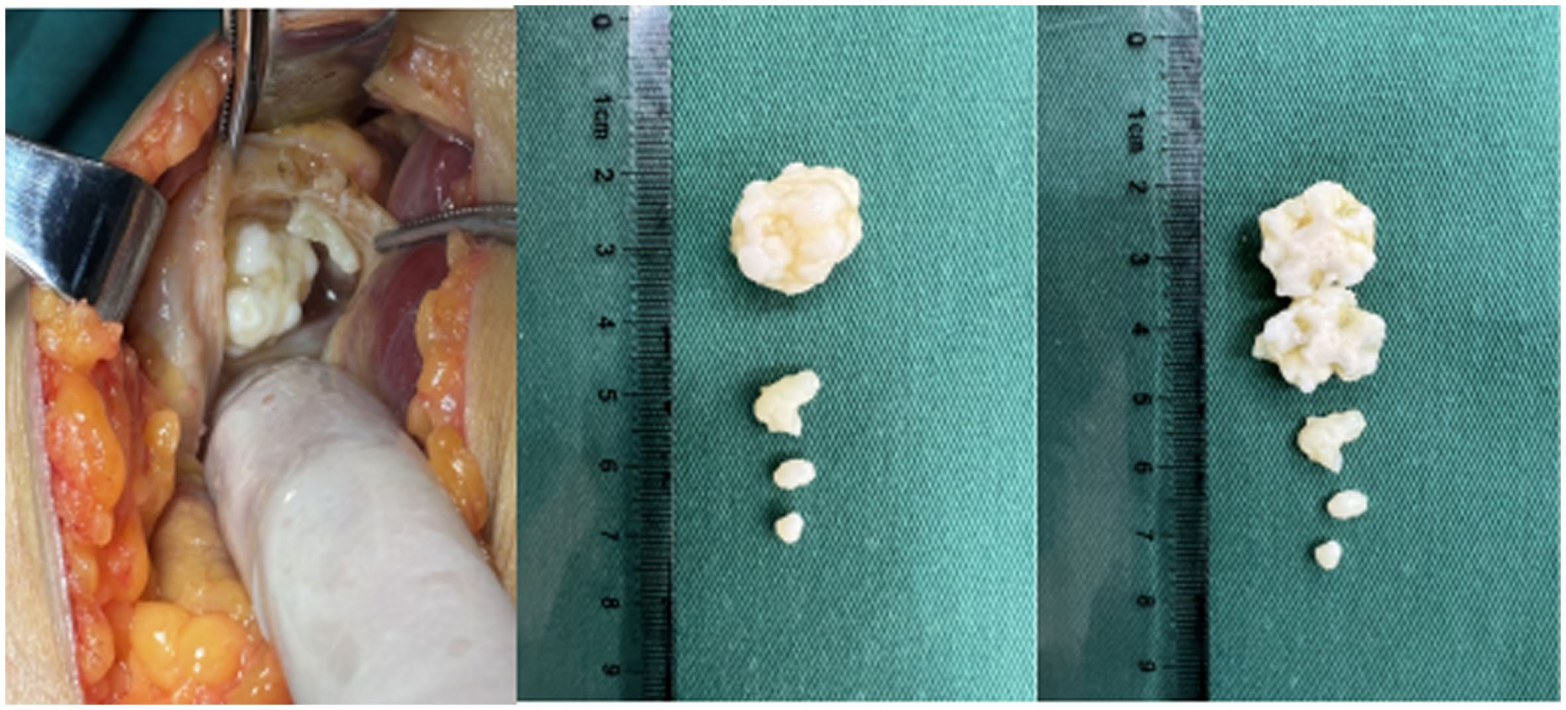
Intraoperative photograph. Incision of the popliteal cyst wall reveals intracystic synovial chondromatosis nodules. The largest nodule has been bisected longitudinally, exposing its internal structure.

Under continuous epidural anesthesia and tourniquet control, the patient was placed prone. A posterior S-shaped surgical approach to the popliteal fossa was utilized to excise the left popliteal cyst and the tumors contained within it. During the procedure, the popliteal vessels and nerves were retracted laterally and protected, while the semitendinosus, semimembranosus, and medial head of the gastrocnemius were retracted medially. By bluntly dissecting the surrounding normal soft tissues, such as tendons adjacent to the cyst wall, and retracting them bilaterally, both the cyst and the tumors were fully exposed. The cyst wall was incised, and four tumors were completely removed. The tumor tissues were found immersed in the fluid of the popliteal cyst, without any apparent connection to the bony structures of the knee joint or the cyst wall tissues, and exhibited a firm texture. The largest tumor measured 2.5 cm × 2.0 cm × 2.0 cm (Figure 5). Following the complete excision of the tumor tissue and the cyst wall of the popliteal cyst, the specimens were sent for pathological examination. After ensuring thorough hemostasis, a negative pressure drainage tube was placed in the wound, followed by layered suturing, and finally, the wound was dressed with a sterile dressing.

### Follow-up and outcomes

**Figure 6:**
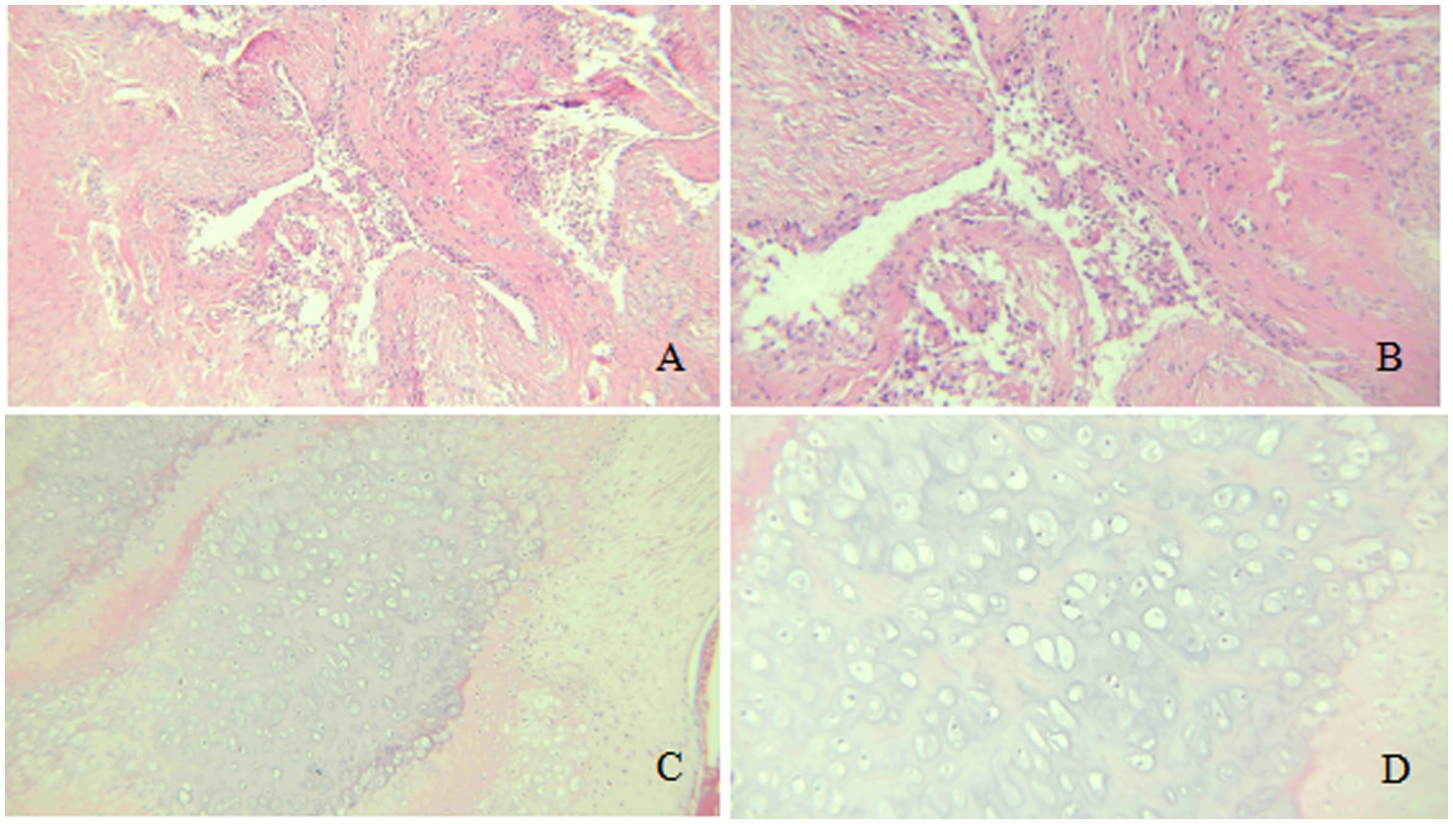
Histopathological analysis (Hematoxylin & Eosin stain). **(A,B)** Cyst wall: Fibrous hyperplasia with hyaline degeneration, chondroid metaplasia (arrowheads), and chronic inflammation (**A**: 100×; **B**: 200×). **(C,D)** Intracystic chondroma: Multilobulated nodule with surface synovial cells (arrow) and underlying hyaline cartilage containing chondrocytes in clusters, showing mild atypia but no mitoses (**C**: 100×; **D**: 200×).

The pathological examination report revealed that the submitted sample from the left popliteal fossa cyst comprises fibrous cyst wall tissue, characterized by hyperplasia of the fibrous tissue within the cyst wall, along with hyaline degeneration and chondroid metaplasia. Numerous foamy histiocytes and chronic inflammatory cell infiltrates are identified in various regions. The sample of the intracystic mass is identified as a multilobulated formation, covered by hyperplastic synovial cells on its surface. Chondroid metaplasia is noted within the nodules, where the cartilage predominantly consists of hyaline cartilage. The chondrocytes are arranged in clusters or trabeculae, with some exhibiting mild atypia; however, definitive mitotic figures are absent, and there is no evidence of infiltration into the surrounding tissues (Figure 6).

#### Final diagnosis

Left popliteal fossa cyst with intracystic synovial chondromatosis. Follow-up at 6 months post-surgery showed no local recurrence of the mass. There was no swelling in the popliteal fossa, no localized tenderness, and the knee’s range of motion for flexion and extension had improved compared to preoperatively. The range of motion for flexion and extension was 0–115°.

## Discussion

Popliteal cyst, also known as Baker’s cyst, was first described by Baker in 1877. This cyst typically occurs in the bursa between the gastrocnemius and semimembranosus muscles, situated between the medial femoral condyle, the semimembranosus tendon, and the medial head of the gastrocnemius. Studies have shown that the cyst wall is composed of synovial tissue and usually communicates with the knee joint cavity. Chronic irritation of the knee joint, meniscal injury, or proliferative arthritis can lead to chronic synovial effusion, resulting in the enlargement and distension of the gastrocnemius and semimembranosus bursae. Clinically, it presents as a palpable mass behind the knee joint. In this study, MRI examination of the patient confirmed the presence of knee joint effusion communicating with the popliteal cyst, accompanied by meniscal and articular cartilage injuries.

Synovial chondromatosis (SC), also referred to as synovial osteochondromatosis, is a clinically rare benign proliferative disorder of the joint synovium. This condition predominantly affects individuals over the age of 40, with a higher prevalence in males, and is most commonly observed in the knee joint, though it rarely affects other joints (4). Histologically, SC is primarily characterized by multiple hyaline cartilage nodules that are closely associated with the synovium; some of these nodules are attached to the synovium, while others may float freely in the synovial fluid. Studies indicate that over 50% of cases exhibit endochondral ossification, although the exact etiology and pathogenesis of this condition remain unclear (5). Most research suggests that factors such as trauma, tumors, and infections may contribute to the development of SC. The knee joint is a high-incidence area, potentially due to long-term weight-bearing activities or pressure that leads to repeated mild injuries to the synovium, or minor trauma that causes the shedding of articular cartilage or cells into the joint cavity, which subsequently proliferate in the synovium. Concurrently, degenerative joint diseases may promote the transformation of synovial cells into chondrocytes (6–8). Furthermore, studies suggest that hyperuricemia may facilitate calcification or oxidative stress through inflammatory mediation, leading to disturbances in calcium and phosphorus metabolism, which accelerates calcium salt deposition and promotes osteogenic differentiation and endothelial barrier dysfunction via the activation of HIF-*α* (12). In this study, the long-term elevation of blood uric acid levels in patients may have resulted in a hyperuricemic environment in the synovial fluid of the popliteal cyst, potentially contributing to the osteogenic differentiation and calcification associated with synovial chondromatosis.

The pathological features of synovial chondromatosis are primarily characterized by chondroid metaplasia of the connective tissue within the synovium, or the inner membrane of the sheath, resulting in the formation of cartilaginous bodies accompanied by synovial hyperplasia (13). Milgram et al. classified the pathological process of synovial chondromatosis into three stages: Stage I represents the active phase of synovial lesions, during which cartilaginous bodies form within the synovium but have not yet become free bodies; Stage II denotes the transitional phase of synovial lesions, wherein synovial hyperplasia and metaplasia gradually lead to the development of pedunculated cartilaginous bodies that are visible to the naked eye, although they have not yet detached; Stage III signifies the quiescent phase of synovial lesions, during which the cartilaginous bodies detach to form free bodies and gradually undergo calcification and osteogenic differentiation under the influence of synovial fluid, resulting in an increase in size over time (14).

X-ray is a commonly used imaging method for diagnosing synovial chondromatosis. In lateral knee X-rays, notable calcification or ossified loose bodies can be observed, which is particularly important for diagnosing patients in Milgram stage III. In this study, the patient’s X-ray images exhibited calcified nodules resembling spherical coral. However, X-ray examinations have difficulty distinguishing synovial chondromatosis from other soft tissue tumors, and there is also uncertainty in diagnosing popliteal cysts. Therefore, further CT and MRI examinations are necessary. CT, through three-dimensional reconstruction, can clearly display the number, location, size, and morphological characteristics of loose bodies. For joint effusion and early knee joint lesions, MRI, with its superior soft tissue signal resolution, can effectively illustrate the degree of synovial hyperplasia, the size of popliteal cysts, and the morphology of intra-capsular loose bodies. These imaging findings provide precise data for determining the number and positional relationships of tumors, offering critical references for the complete intraoperative removal of lesions and reducing the postoperative recurrence rate (15).

The imaging findings of calcification or ossification within a popliteal cyst are extremely rare, and the occurrence of synovial chondromatosis within the cyst is even rarer. Khadka et al. reported a case of synovial chondroma extending from a popliteal cyst into the knee joint. They performed anterior arthroscopic surgery combined with posterior cyst removal to excise extensive, irregular synovial chondromas and popliteal cysts (9). Sankepally et al. identified a patient with bilateral popliteal cysts and knee joint synovial chondromatosis using X-ray and color Doppler ultrasound, which revealed typical chondroma-like free bodies on ultrasound (11). In this case, the patient’s synovial chondromatosis exhibited a spherical coral-like appearance, a phenomenon not previously reported in the literature. This may be attributed to the shedding of cartilage or cells through the posterior fissure of the knee joint cavity into the popliteal cyst during the degenerative process of the knee joint. Poor long-term control of hyperuricemia resulted in a high uric acid environment within the popliteal cyst, which accelerated the osteogenic differentiation and calcification of the synovial chondromatosis. Additionally, the synovial lining of the popliteal cyst may have been influenced by the synovial lesions associated with the synovial chondromatosis present within the knee joint cavity (11). During knee flexion and extension, the synovial chondroma within the popliteal cyst was compressed and molded during its growth, led to the twisting of branching differentiation in various directions, which results in a spherical coral-like appearance. Currently, the primary treatment for popliteal cysts combined with intrasynovial chondromas is surgical excision. In this study, after the excision of the popliteal cyst and intrasynovial chondroma, the patient experienced significant relief from local pain, and the range of motion in knee flexion and extension was largely restored. Studies indicate a 23% recurrence rate after complete resection of synovial chondromatosis, with a malignancy incidence of 1% to 6%. Multiple recurrences raise the risk of malignancy, necessitating long-term follow-up and observation.

In conclusion, for patients exhibiting knee pain and popliteal swelling, along with imaging findings that indicate a mass within the popliteal cyst, the diagnosis of a popliteal cyst combined with synovial chondromatosis should be considered. It is important to note that the occurrence of intra-cystic spherical coral-like synovial chondromatosis within a popliteal cyst is exceedingly rare. Currently, the primary treatment method involves the surgical excision of the popliteal cyst synovium along with the intra-cystic synovial chondromatosis.

## Data Availability

The raw data supporting the conclusions of this article will be made available by the authors, without undue reservation.
